# Water-Borne Diseases and Extreme Weather Events in Cambodia: Review of Impacts and Implications of Climate Change

**DOI:** 10.3390/ijerph120100191

**Published:** 2014-12-23

**Authors:** Grace I. Davies, Lachlan McIver, Yoonhee Kim, Masahiro Hashizume, Steven Iddings, Vibol Chan

**Affiliations:** 1 Faculty of Medicine, Nursing and Health Sciences, Monash University, Melbourne, Victoria 3800, Australia; E-Mail: gidav2@student.monash.edu; 2 National Centre for Epidemiology and Population Health, Australian National University, Canberra, A.C.T. 2601, Australia; 3 Institute of Tropical Medicine, Nagasaki University, Nagasaki 852-8523, Japan; E-Mails: yoonhee@nagasaki-u.ac.jp (Y.K.); hashizum@nagasaki-u.ac.jp (M.H.); 4 World Health Organization, Cambodia Country Office, Phnom Penh, Cambodia; E-Mails: iddingss@wpro.who.int (S.I.); chanv@wpro.who.int (V.C.)

**Keywords:** flood, drought, extreme weather event, climate change, Cambodia, health, water-borne disease, diarrhoea

## Abstract

Cambodia is prone to extreme weather events, especially floods, droughts and typhoons. Climate change is predicted to increase the frequency and intensity of such events. The Cambodian population is highly vulnerable to the impacts of these events due to poverty; malnutrition; agricultural dependence; settlements in flood-prone areas, and public health, governance and technological limitations. Yet little is known about the health impacts of extreme weather events in Cambodia. Given the extremely low adaptive capacity of the population, this is a crucial knowledge gap. A literature review of the health impacts of floods, droughts and typhoons in Cambodia was conducted, with regional and global information reviewed where Cambodia-specific literature was lacking. Water-borne diseases are of particular concern in Cambodia, in the face of extreme weather events and climate change, due to, *inter alia,* a high pre-existing burden of diseases such as diarrhoeal illness and a lack of improved sanitation infrastructure in rural areas. A time-series analysis under quasi-Poisson distribution was used to evaluate the association between floods and diarrhoeal disease incidence in Cambodian children between 2001 and 2012 in 16 Cambodian provinces. Floods were significantly associated with increased diarrhoeal disease in two provinces, while the analysis conducted suggested a possible protective effect from toilets and piped water. Addressing the specific, local pre-existing vulnerabilities is vital to promoting population health resilience and strengthening adaptive capacity to extreme weather events and climate change in Cambodia.

## 1. Introduction

**Figure 1:**
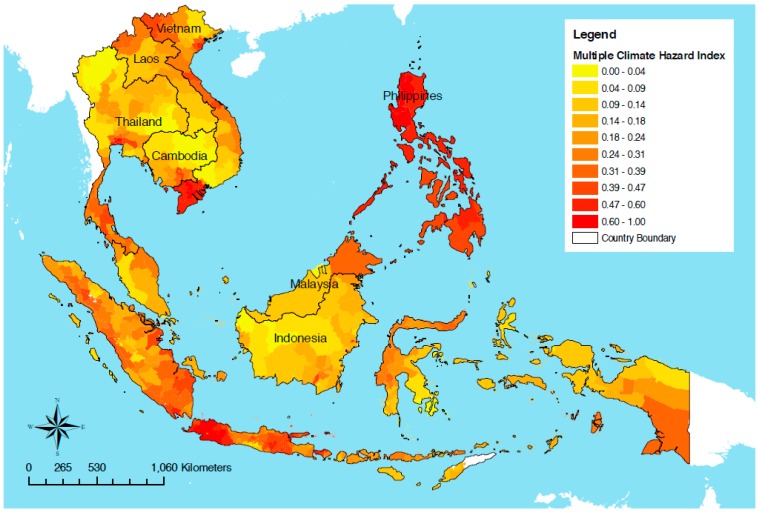
Map of vulnerability to climate change in Southeast Asia, taking into account exposure to climate hazards (tropical cyclones, floods, landslides, droughts and sea level rise), sensitivity and adaptive capacity. 0 indicates lowest vulnerability, 1 indicates highest vulnerability. Source: Reproduced with permission from Yusuf and Franciso [1].

Southeast Asia is identified as a ‘climate change hotspot’ and Cambodia is considered one of the most vulnerable countries in Southeast Asia to the impacts of climate change (Figure 1) [1,2].

Cambodia is identified as having ‘extreme’ vulnerability to climate change, ranking eighth out of 193 countries in Maplecroft’s Climate Change Vulnerability Index in 2014, based on a composite of exposure to extreme weather events (predominantly floods and droughts), sensitivity and adaptive capacity [1,3]. Cambodia has a tropical monsoon climate comprising annual dry and wet seasons [4]. The wet season usually occurs from May to November, with prominent rainfall (85% of annual rainfall) from the southwest monsoon [4,5,6]. Much of Cambodia is a floodplain, with 85% of the country’s land within the lower Mekong basin [7]. During the wet season, tropical storms, heavy monsoonal rains, and run-off from the northern mountains cause seasonal flooding and overflow of the Mekong River, its tributaries and the Tonle Sap River [5]. This annual flooding provides crucial inundation of agricultural land along the Mekong and Tonle Sap Rivers for one to four months each year, replenishing the soil after the dry season, enabling rice harvest and nutrition to fisheries, vital for food security and income in Cambodia [8,9]. Climate change has the potential to increase the frequency and intensity of flooding and/or drought, both of which already cause severe hardship to communities in Cambodia [9,10,11,12,13,14]. 

Over 80% of Cambodia’s population of 15.14 million live and work in the rural floodplain regions of the country’s 24 provinces [9,14]. The health impacts of floods and droughts in Cambodia are of substantial concern, given the pre-existing vulnerabilities of the population and low adaptive capacity of the population. These vulnerabilities relate to, for example, widespread poverty, poor health and malnutrition; settlements in flood-prone areas; reliance on agriculture for food security and income; low education levels; inadequate warning systems; and resource, governance and public health limitations [1,3,15].

Of particular concern in Cambodia is the potential impact of flooding and drought on water-borne diseases, primarily diarrhoeal disease (*i.e*., viral and bacterial gastroenteritis, dysentery, cholera and other manifestations of gastrointestinal infections) [16]. Diarrhoea is the second most common inpatient and outpatient diagnosis and second leading cause of death for children under five in Cambodia [17,18]. Seventy-five percent of the rural population lack access to improved sanitation means. The majority of the rural population (66%) openly defecates, with a small proportion (9%) having access to pit latrines (without a slab or platform), hanging latrines and bucket latrines [19]. Contrastingly, 82% of the urban population have access to improved sanitation facilities—flush or pour flush to a piped sewer system, septic tank, pit latrine, ventilated improved pit latrine and pit latrine with slab or composting toilet [19]. Furthermore, thirty-four percent of the rural population rely on unimproved water sources—unprotected dug wells or springs, carts with small tanks or drums, bottled water or surface drinking water from rivers, dams, lakes, ponds or streams [19]. Ninety-four percent of the urban population have access to improved water sources—piped water on premises, public taps or standpipes, tube wells or boreholes, protected dug wells or springs and rainwater collections [19]. This disparity highlights the amplified vulnerability of the rural population to water-borne illness.

Extreme weather events and climate change are two major drivers of water-borne diseases [20]. Extreme weather events are associated with water-borne disease outbreaks in Asia and elsewhere in the world [21] and diarrhoea remains a leading cause of death from natural disasters globally, particularly in low-income countries [22]. The high baseline burden of disease and existing risk factors for water-borne diseases in Cambodia indicate that the added burden of extreme weather events anticipated via climate change is likely to present a key health challenge to this developing nation. 

Given that climate change is predicted to increase the frequency and intensity of extreme weather events in Cambodia, an understanding of the water-borne health impacts and associated vulnerabilities is required to evaluate risk, direct planning, prioritize resources, and develop targeted responses to strengthen resilience and adaptive capacity of this highly vulnerable population.

The aims of this paper are therefore twofold:

1. To synthesize the literature on the water-borne health impacts of floods, droughts and typhoons in Cambodia.
2. To explore the relationship between flooding and diarrhoeal disease in Cambodian children.

## 2. Experimental Section 

### 2.1. Literature Review of Extreme Weather Events (Floods, Droughts and Typhoons) and Water-Borne Health Impacts in Cambodia

Quantitative and qualitative data of the floods, droughts and typhoons in Cambodia from 1991–2013 were collated (Table 1). This time period was chosen to give the most comprehensive possible overview of the extreme weather events in Cambodia over the period for which data is available. Prior to this time, data is limited due to the dysfunction of the civil conflict. Types of data reviewed for each extreme event included: time of year; duration of event; provinces affected; number of people killed, injured, affected and displaced; number of homes affected; area of agricultural land affected; documented health impacts, particularly water-borne illnesses; and estimated total US$ damages. EM-DAT (Emergency Events Database maintained by the Centre for Research on the Epidemiology of Disasters (CRED)), ReliefWeb (specialized digital service of United Nations Office for the Coordination of Humanitarian Affairs (OCHA)) and International Federation of the Red Cross (IFRC) reports were the principal sources of this information.

Data from media articles including those sourced from the Asian Disaster Reduction Centre (ADRC) were also included. Multiple sources were utilized to crosscheck and corroborate results. 

A literature search using Medline was then conducted to produce a qualitative review of the water-borne health impacts of floods and droughts in Cambodia, or regionally, where Cambodia-specific literature was lacking. Search terms included ‘drought’ OR ‘flood’ AND ‘health’ AND ‘Cambodia’ OR ‘Asia’. Primary research from the region; review articles including systematic reviews; and institutional, governmental and international reports were included.

### 2.2. Flood Events and Diarrhoeal Disease in Cambodia

To evaluate an association between floods and diarrhoeal disease, flood events and diarrhoeal disease cases in 16 Cambodian provinces were studied over a 12-year time period (see Table 1). Floods were selected as the most appropriate extreme event to review in this analysis, due to data availability and completeness and the applicability of a uniform definition, both of which were more problematic when applied to droughts.

Sixteen provinces (out of 24) were selected based on data quality: Banteay Meanchey, Battambang, Kampong Thom, Kampot, Koh Kong, Kratie, Pailin, Phnom Penh, Pursat, Prey Veng, Ratanakiri, Siem Reap, Stung Treng, Svay Rieng, Kampong Cham, and Preah Sihanouk. 

**Table 1:** A summary of the impacts of extreme weather events (floods, droughts and typhoons) in Cambodia from 1991–2013. Adapted from: [23,24].

Year	Event (Month/s)	No. of provinces affected	Impacts
1991	Flood (August)	10	100 people died, 900,000 people affected 243,000 hectares agricultural land affected, $150,000,000US estimated damages
1994	Drought (June)		5,000,000 people affected, $100,000,000US estimated damages
	Flood (July)	6	506 people killed, 12,000 people displaced
1996	Flood (September)	10	59 people killed, 1,372,410 people affected 30,577 hectares agricultural land affected, 584,693 people affected by food shortage $1,500,000US estimated damages
1997	Typhoon (November)	1	23 people killed, 200 people missing
1997–1998	Drought (“Late 1997–Early 1998”)		Food shortages mid-year
1999	Flood (July–August)	7	4 people killed, 535,904 people affected, 8100 people displaced, 7000 homes affected 17,732 hectares agricultural land affected, $500,000US estimated damages
	Flood (November)	6	25,847 families affected, 3561 homes affected 9990 hectares agricultural land affected
2000	Flood (July–November)	22	347 people killed (80% children), 3,448,629 people affected, 387,000 people displaced 325,043 homes affected 421,569 hectares agricultural land affected $156,655,500US estimated damages
2001	Drought (“Most of the year”)	12	530,844 people affected by food shortage
	Flood (August–October)	18	62 people died (70% children), 2,121,952 people affected, 508,666 people displaced 201,371 hectares agricultural land affected, 945,665 people affected by food shortage $16,900,000US estimated damages, (total 2001 drought & flood: $36,000,000US) **Increase in diarrhoeal disease**
2002	Drought (January–August)	24	2,660,000 people affected, 30,000 forced migration 246,643 hectares agricultural land affected, 1,000,000 people affected by food shortage $38,000,000US estimated damages
	Flood (August)	6	29 people killed, 1,470,000 people affected, 450,000 people displaced 2731 hectares agricultural land affected, 470,000 people affected by food shortage $100,000US estimated damages
2004–2005	Drought (October 2004–April 2005)	14	2,000,000 people affected, 1,000,000 people affected by food shortage 520,000 hectares of agricultural land affected, $21,000,000US estimated damages
2006	Tropical Storm Prapiroon, Flood (August–September)	9	13 people killed, 33,000 people displaced, 263 houses affected 17,515 hectares of agricultural land affected
2007	Flood (June)	1	
	Tropical Storm Pabuk, Flood (August)	8	5 people killed, 160,000 people affected 8000 hectares agricultural land affected $1,000,000US estimated damages
2008	Flood (August)	1	
	Drought (September)	1	
2009	Drought (August)	7	79,000 hectares agricultural land affected
	Typhoon Ketsana, Flood (September–October)	14	43 people killed, 67 people injured, 180,000 people affected, 6210 families displaced 10,000 homes affected 57,000 hectares agricultural land affected, 48,000 families affected by food shortage $131,996,415US estimated damages **Increase in diarrhoeal disease**
	Typhoon Mirinae (November)	1	2 people killed, 4 people injured
2009–2010	Drought (November 2009–July 2010)		
	Flood (October–November)	8	8 people killed, 5 people injured, 9726 families affected 33,096 houses affected 18,527 hectares agricultural land affected $70,000,000US estimated damages
2011	Flood (August–November)	18	247 people killed, 23 people injured, 1,640,023 people affected, 214,000 people displaced, 270,371 houses affected 423,449 hectares agricultural land affected, 15% households severely food insecure $521,000,000US estimated damages **Increase in diarrhoeal disease**
2012	Tropical Storm Pakhar (March–April)	1	5 people injured 145 houses affected
	Drought (July–August)	14	146,140 hectares agricultural land affected
	Flood (September–October)	8	27 people killed, 14,322 families affected, 4057 families displaced 12,274 houses affected, 16,510 hectares agricultural land affected
	Tropical Storm Gaemi (October)	7	
2013	Flood (August)	4	13 people killed, 2592 families affected, 450 families displaced, 230 houses affected 20,000 hectares agricultural land affected
	Flood, Typhoon Usagi, Tropical Storm Krosa (September–October)	21	188 people killed, 29 people injured, 1,735,828 people affected, 144,044 people displaced 240,195 houses affected 384,846 hectares agricultural land affected $1,000,000,000US estimated damages **Increase in diarrhoeal disease**

Monthly number of diarrhoeal disease cases in children aged 14 and under were sourced from the Cambodian Ministry of Health (MoH)—specifically, weekly notification data from all MoH health facilities (including outpatient clinics and hospital inpatients)—and were stratified by province. This age group was chosen due to the high vulnerability of children to diarrhoeal disease and high child mortality and morbidity from diarrhoeal disease in Cambodia [17,25]. Flood events were coded as a binary variable (*i.e*., flood *versus* no flood). Unfortunately, no mortality data was available for the purposes of analysis as part of this project.

The diarrhoeal data was available from 2001 to 2012 and this formed the study period, except in two provinces where temperature data were limited to 6 years (in Pailin) and 4 years (in Ratanakiri) (see Table 2).

Local weather data were provided by the Cambodian Ministry of Water Resources and Meterology (MoWRAM), which consisted of monthly mean temperature and total precipitation from the same time period as the diarrhoea cases. Two outliers of mean temperature in Kampot and Svay Rieng were excluded.

The flood events were defined using the databases of EM-DAT (http://www.emdat.be/result-country-profile) and ReliefWeb (http://reliefweb.int/country/khm). The flood events identified in Table 1 were transformed to the binary variable with 0 (non-event) or 1 (event) in our statistical model.

Time-series analysis under quasi-Poisson distribution was applied. Two statistical models were used to analyze the estimated flood effect on diarrhoeal disease: (1) a model adjusting for seasonality and long-term trends (the simplest) and (2) a model adding adjustment for mean temperature to the simplest model. The final model is given as follows:

Log E[Y_t_] = α + β_1_∙Flood_t–l_ + β_2_∙T_MA0-3_ + ns(t,df_t_) + I(y) (1)

where Y*_t_* is the observed diarrhoea cases on month *t*; α is the intercept; Flood*_t–l_* is binary variable on month *t* at lag of *l* months—we considered single-month lags of flood on the same month (*l*_0_), the previous month (*l*_1_), two months previous (*l*_2_), three months previous (*l*_3_); β_1_ is coefficient for Flood*_t–l_*; T_MA0-3_ is moving average over the current and previous three months of monthly mean temperature; β_2_ is coefficients for T_MA0-3_; ns(*t*,df*_t_*) is natural cubic spline of time (*t*) with df*_t_* = 4/year to adjust for long-term trend and seasonality of diarrhea; I(y) is an indicator of year.

Linear models were used to examine an association between the effect of flood on diarrhoea and hygiene and sanitation status. Using meta-regression analysis, the effect of flood on diarrhoea was examined according to hygiene and sanitation status—defined as the proportion of households with a toilet or piped water for drinking—in each province, derived from analysis conducted in a earlier phase of climate change and health project work in Cambodia [16]. Phnom Penh, the capital city, was excluded from this analysis as it is urbanized and has much higher rates of households with a toilet (any type of “improved” sanitation, *i.e*., excluding pit latrines) (87.3%) and pipe water drinking (77.0%) compared with the 80% of Cambodia’s population that lives rurally (only 25% of the rural population have access to improved sanitation facilities) [19].

**Figure A1:**
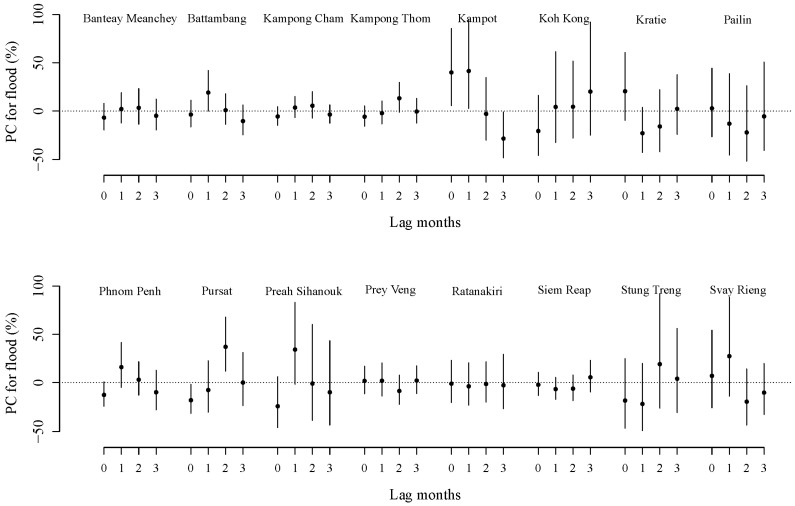
Flood effects on diarrhoea in 16 provinces adjusting for rainfall, temperature, long-term trend and seasonality.

**Figure A2:**
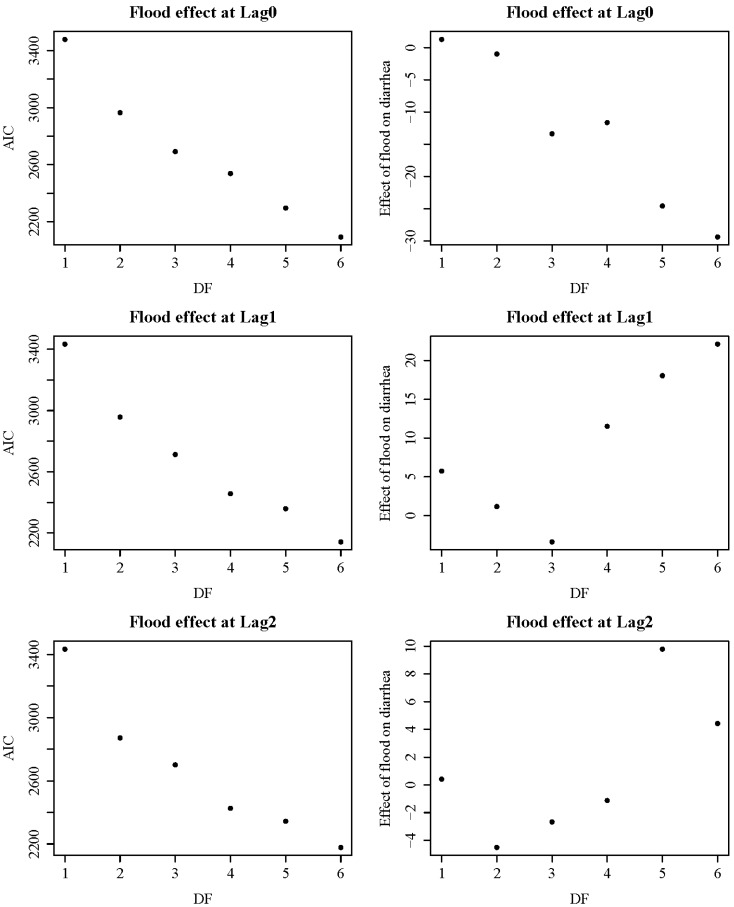
AIC and flood effect estimates on diarrhoea according to changing degree of freedom (DF) from one to six. We selected four degrees of freedom, showing smaller AIC and relatively stable effect estimate of flood on diarrhoea.

**Figure A3:**
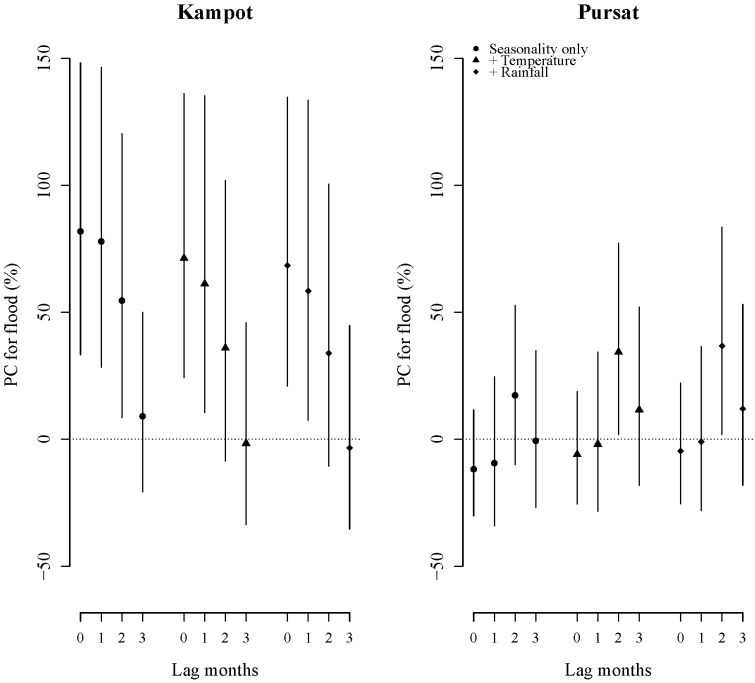
Flood effects on diarrhoea by distributed lag models in two provinces.

A sensitivity analysis was also conducted, as flood events can be an intermediate factor between rainfall and diarrhoea incidence. An additional model was then compiled, adjusting for rainfall, in addition to a final model, to examine whether the effects of flood on diarrhoea were consistent (Figure A1). Distributed lag models were also used to confirm the consistency of the results (Figure A1, Figure A2 and Figure A3) and the results were compared with those from single-lag models.

The distributed lag model is given as follows:

Log E[Y*_t_*] = α + β_1_∙Flood*_t_* + β_2_∙Flood*_t_*_–1_ + β_3_∙Flood*_t_*_–2_ + β_4_∙Flood*_t_*_–3_ + β_5_∙T_MA0-3_ + ns(*t*,df*_t_*) + I(y) (2)

The following formula was used to calculate the percent change (PC) of the flood effect in the final model: $\text{PC}=((e^{\beta })-1)\times 100$ . To set a degree of freedom for the term of seasonality and long-term trends, the number of degrees of freedom were changed from one to six, and the AIC (Akaike’s information criterion) of the models were compared (Figure A2) [26]. Because AIC was not available under a quasi-Poisson regression, Poisson regression was used. A robustness of effect estimates of flood and AIC were visually inspected in Figure A2, and four degrees of freedom was finally selected as the best model. R software (version 3.1.0, R Development Core Team 2009) was used in all analyses. The statistical packages in R we used were ‘splines’ and ‘tsModel’.

## 3. Results 

### 3.1. Extreme Weather Events (Floods, Droughts and Typhoons) in Cambodia 

A summary of floods, droughts and typhoons in Cambodia from 1991–2013 is shown in Table 1. 

Each year, floods of varying intensity affect Cambodia [6]. In the past, Cambodia’s annual wet season floods were mainly beneficial, providing vital inundation of agricultural soil after the dry season [9]. Devastating floods used to occur every five years or more (1961, 1966, 1978, 1984) [25]. Recently, floods classified as ‘disasters’ on the Reliefweb database appears to have become more frequent in Cambodia, occurring in 1991, 1994, 1996, 1999, 2000, 2001, 2002, 2006, 2009, 2010, 2011 and 2013 [23]. Furthermore, some floods have been uncharacteristically intense. The 2000 floods were the worst in 70 years, affecting 3,448,629 people in 22 provinces [27]. The 2011 floods were then classified as the “worst since 2000”, affecting 18 provinces and 1,640,023 people [28].

Weaker, shorter wet seasons have also been noted in some years, resulting in droughts in 1991, 1992, 1994, 1997, 1998, 2001, 2002, 2004, 2005, 2008, 2009, 2010 and 2012 [24]. The droughts of 2002 and 2004—5 were classified the worst in 20 and 50 years respectively affecting all 24 and 14 Cambodian provinces respectively [29]. 

### 3.2. Review of Water-Borne Diseases and Extreme Weather Events in Cambodia 

These results are a summary of the reviewed literature. Diarrhoeal disease is the predominant water-borne disease identified following severe flooding in the past two decades in Cambodia, particularly affecting children under five [6,30,31,32]. Other water-related diseases identified following floods in Cambodia include ear, nose and throat infections, wound infections, dermatitis and conjunctivitis [8,25,33].

Increases in diarrhoea have been noted since 2001 in the two worst flood-affected provinces (Prey Veang, Kampong Cham) and in some ‘safe’, evacuation areas (Safe areas: traditional places of refuge on higher ground such as pagodas, schools and roads) [31]. Diarrhoeal disease also occurred following the 2009 typhoon and 2011 and 2013 floods in Cambodia [32,34,35]. Following the severe Cambodian floods in 2011, an increase in diarrhoea to epidemic levels occurred in three provinces (Banteay Meanchey, Oddar Meanchey, Kampong Thom) and 22% of children (0–59 months) suffered from diarrhoea in early 2012 [28,35]. Those from households that were poor, moderately-severely flood-affected, had untreated drinking water, non-improved sanitation facilities, lacked soap, lacked maternal education or relied on uncovered community wells for drinking water were found to be at greatest risk [35,36].

Drought has also been associated with an increased risk of water-borne diseases such as diarrhoea in Cambodia [25]. Reduced water availability can lead to use of unsafe water sources and often forces people to travel greater distances to access water [25]. Furthermore, malnutrition—which is common in drought conditions—is a known risk factor for transmission of infectious diseases, including diarrhoeal illness. 

#### Other Water-Related Diseases

Other water-related diseases (Water-related disease: transmitted via contact, ingestion, inhalation, skin penetration, altered geographic range of vectors or vertebrates) in Cambodia, which may be susceptible to the effects of climate change, include leptospirosis, typhoid fever, melioidosis, schistosomiasis, viral hepatitis and arsenicosis [16,37,38,39,40].

Leptospirosis is spread by water contaminated by rodent urine and is considered endemic in Thailand, Cambodia, Laos and Vietnam. It typically occurs in seasonal peaks during the rainy season, with many outbreaks specifically related to floods [40,41]. A study of two districts in Cambodia showed significantly higher rates of rodent infection of leptospirosis in the wet season [40], which corresponds with the observed season increase of human leptopsirosis infection during the wet season. 

Typhoid fever is another common water-related disease following severe flooding in the past two decades in Cambodia [6,30,31,32,33], although there is little recorded outbreak or incidence data. Increases in typhoid fever related to floods have been reported in low and middle-income countries, namely in some Asian and African countries [42,43,44,45].

Melioidosis is a serious and often fatal infection caused by *Burkholderia pseudomallei*, which is found in water and soil and is endemic in Southeast Asia and Northern Australia [39,46]. Melioidosis cases tend to increase during the wet season [39]. The intensity of rainfall is an independent predictor of melioidosis admission with pneumonia, septic shock and death [39].

Schistosomiasis is spread by contact with water containing parasites from snails. Flooding in Asia is thought to increase the risk of schistosomiasis outbreak [47] and may alter the range of schistosomiasis, by potentially spreading snails to previously unaffected areas or re-introducing them to previously affected areas [48].

Arsenicosis and viral hepatitis are also diseases related to consumption of contaminated water and are known to exist in Cambodia.

Water-borne and water-related conditions also have wider health consequences. A lack of private and secure sanitation facilities can undermine the physical and mental health and safety of girls and women in congested living spaces as often occurs in evacuation centres [33]. Children affected by chronic diarrhoea are also at increased risk of poor growth, malnutrition, cognitive deficits and poorer schooling outcomes [33,49,50].

### 3.3. Floods and Diarrhoeal Disease in Cambodian Children 

The mean number of diarrhoea cases during months with and without floods; total number of months that experienced flooding; and local weather (rainfall and temperature) of the 16 Cambodian provinces over the study period are displayed in Table 2.

**Table 2:** Characteristics of the studied provinces in Cambodia. IQR indicates interquartile range.

Provinces	Study Period	Mean Monthly No. of Diarrhoea Cases in Children up to 14 Years ^a^		No. of Months Affected by Flooding ^b^	Rainfall ^c^ (mm)		Mean Temperature ^c^ (ºC)
		Non-flood	Flood		Median (IQR)	Max	
Banteay Meanchey	2001–2012	828	871	13	88.5 (149.3)	452.2	28.4
Battambang	2001–2012	747	845	14	82.6 (142.8)	353.3	28.3
Kampong Thom	2001–2012	620	569	15	99.4 (180.3)	497.2	27.6
Kampot	2001–2012	243	237	11	131.4 (209.1)	629.1	28.1
Koh Kong	2001–2012	101	90	6	197.5 (354.4)	1600.8	27.7
Kratie	2001–2012	267	380	10	126.7 (221.7)	537.8	28.5
Pailin	2007–2012	135	109	6	106.2 (128.3)	374.8	27.6
Phnom Penh	2001–2012	650	705	12	98.0 (168.1)	410.3	28.9
Pursat	2001–2012	180	219	11	105.6 (165.9)	398.6	28.4
Prey Veng	2001–2012	1719	1587	8	108.8 (169.0)	544.8	28.3
Ratanakiri	2004–2008	385	434	3	57.6 (263.3)	746.7	26.8
Siem Reap	2001–2012	885	1517	10	90.5 (197.1)	512.8	28.4
Stung Treng	2001–2012	54	45	8	74.5 (215.2)	552.8	28.4
Svay Rieng	2001–2012	507	524	7	127.5 (211.1)	499.1	28.2
Kampong Cham	2001–2012	2319	1990	10	80.5 (101.0)	170.0	28.2
Preah Sihanouk	2001–2012	152	135	3	86.5 (81.5)	182.0	28.0

Notes:^ a ^Sourced from: Cambodian Ministry of Health (MoH). ^b ^Sourced from: Figure 1. ^c ^Sourced from: Cambodian Ministry of Water Resources and Meterology (MoWRAM).

**Figure 2:**
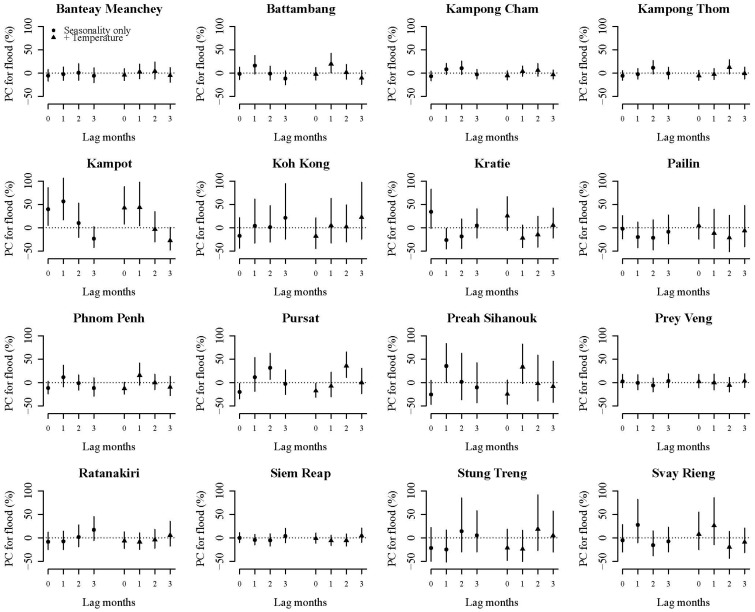
Flood effect on diarrhoeal disease cases in 16 Cambodian provinces. ‘PC’ indicates a percent change of the flood effect on diarrhoea in each province, meaning the change in diarrhoeal disease cases corresponding to flood events after adjusting for temperature, long-term time trend and seasonality. ‘Seasonality only’ indicates the simplest model adjusting for seasonality, long-term trends, and year. ‘+ Temperature’ indicates the model adding adjustment for mean temperature to the simplest model. In Kampot, the significant effect of flood on diarrhea was observed at lag0 and lag1; and the effect of flood decreased on the increase in lag months. In Pursat, the significant effects of flood on diarrhea was observed at lag2, meaning that flood events two months ago influenced the increase in diarrhoea cases observed, whereas a protective effect of flood on diarrhoea cases was observed at lag0.

Flooding was significantly associated with diarrhoeal disease incidence in two provinces, Kampot and Pursat (effects at lag0 and lag1 in Kampot and at lag2 in Pursat). These results were consistent when applying distributed lag models. The impact of flooding on diarrhoeal incidence in the other Cambodian provinces was varied (Figure 2). There was also a significant effect of flood on diarrhoeal disease cases in Battambang and Preah Sihanouk; but they were inconsistent when applying distributed lag models. Higher prevalence of households with toilet and piped water are likely to be protective against flood effects, although there was no statistical significance (β = −0.64, *p* = 0.18 for toilet use; β = −0.92, *p* = 0.33 for piped water use).

## 4. Discussion

### 4.1. Key Findings

To our knowledge, this is the first review of the water-borne health impacts of extreme weather events in Cambodia. Incidence and risk of water-borne diseases appears to increase with flooding, particularly in the most flood-affected provinces. 

This study was also the first analysis of long-term diarrhoeal disease incidence and flood events in Cambodia and showed a significant association between flooding and increased diarrhoea cases in children in two Cambodian provinces, Kampot and Pursat—provinces which are considered to be, respectively, the seventh and eighth most vulnerable provinces in Cambodia to climate change [1]. 

Although causation is difficult to ascertain, this association was expected and is consistent with disaster reports of increased diarrhoeal incidence following severe flooding in Cambodia and with a recent study of hydraulic modeling, which found that the annual average risk of water-borne disease from medium-sized floods is 0.21 in Cambodia [8]. Reduced access to health services during floods and compromised power, leading to inability to boil or treat water during floods, are likely key risk factors. This finding is also consistent with the large body of research linking flooding to increased risk of diarrhoeal disease and outbreaks in Asia and internationally [21,38,42,43,44,45,51,52,53,45,51].

The heterogeneous associations observed in the remaining 14 provinces studied could be due to variable contamination of water sources during or after flooding, and the differing capacities or behaviours of households to manage such health risks. Furthermore, between-provincial differences in geographical and geological conditions such as water absorption of soil and underground water levels, baseline sanitation and hygiene levels, and sewage and drainage infrastructure could have contributed to the different effect estimates. 

Common aetiologies of paediatric diarrhoeal diseases in Cambodia include *Escherichia coli* and Rotavirus [54], with cholera occurring in relatively frequent epidemic cycles and strongly linked to temperature, rainfall and other environmental changes [55]. A recent systematic review identified *Vibrio* spp. (namely *V.Cholera*) and *Leptospira* spp., followed by *E.coli*, as the most common pathogens in water-borne disease outbreaks following flooding, with outbreaks most common in Asia [21,42,43,44,45]. Flooding in China in 2007 saw a significant rise in diarrhoea cases, with young children and the elderly most vulnerable [56]. In August 2007, 21,401 cases of diarrheal illness were treated in a Bangladesh hospital due to severe flooding, more than tripling the number of cases treated in August 2006 [57]. Following the Pakistan floods in 2010, diarrhoea was a leading cause of illness, accounting for 17% of medical consultations in the worst-affected province [58]. Although droughts incur significant social and health burdens in Cambodia, it was not possible to quantify their health effects in this project. 

### 4.2. Vulnerabilities of the Cambodian Population

A concerning aspect of floods and droughts in Cambodia is the specific and prominent pre-existing vulnerabilities of the population. Extreme weather events in Cambodia disrupt basic health needs of water, food, shelter and medications and may amplify existing poverty and related health challenges, thereby increasing the population’s vulnerability to subsequent extreme weather events and leading to a vicious cycle of diminishing resilience and deepened vulnerability. 

**Figure 3:**
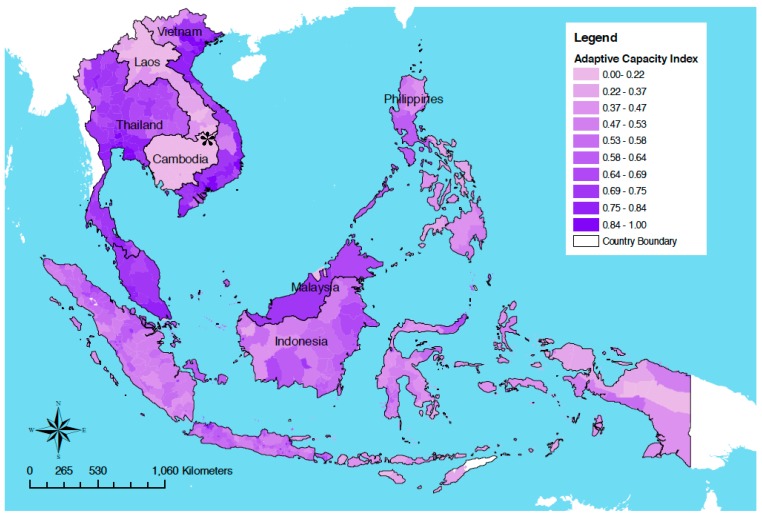
Adaptive capacity to climate change in Southeast Asia. Cambodia (*) and Laos have the lowest adaptive capacity in the region. Source: Reproduced with permission from Yusuf and Franciso [1].

Poverty and vulnerability to disaster are closely related. Cambodia is one of the poorest countries in the world and among the most vulnerable globally to climate change, predominantly due to low adaptive capacity (Figure 3) [1,59,60]. Adaptive capacity refers to the capability of a system or population to adapt to climatic stimuli or their effects or impacts and factors include socio-economic factors, technology and infrastructure. It is thus a determinant of overall vulnerability, but is a distinct concept requiring separate consideration in the context of building resilience to disasters and climate change.

The provinces of Rattanakiri, Mondulkiri, Preah Vihear and Kampong Speu and Takeo are considered to be those most vulnerability to climate change impacts in Cambodia [1]. The characteristics leading to their significant vulnerability provide insight into Cambodia’s low adaptive capacity. Rattanakiri and Mondulkiri have the lowest human development and highest poverty indices in Cambodia; Kampong Speu and Takeo are at heightened risk of flooding; and Takeo is especially threatened by sea level-rise [1]. As Cambodia is an agrarian country, the majority of the population live in the low-lying floodplain regions, which are ideal for rice production but highly susceptible to flooding [9,61].

Extreme weather events may exacerbate existing health challenges. Diarrhoea causes significant morbidity and mortality among children under 15 and the elderly in Cambodia [62,63]. Cambodia has the highest mortality rates (14% compared with 4.6% average) in Southeast Asia of children under the age of five [8], which strongly correlate with sanitation conditions [25,64]. High baseline burdens of infectious disease (e.g., diarrhoea, respiratory infections, dengue fever) and malnutrition (45% of children under five suffer from chronic malnutrition) in Cambodia further compromise immunity [15,16,25,33,49,59,60,62,64,65,66]. Children are physiologically more susceptible to diarrhoeal disease than adults and more likely to die or be severely affected by flooding and its health consequences [67]. 

Contaminated drinking water is a key risk factor for water-borne disease outbreaks during floods and outbreak risk is highest in developing countries with unimproved water sources [68]. Inundation of human settlements, infrastructure damage and population displacement with flooding in Cambodia may reduce clean water for drinking, cooking, irrigation and sanitation, highlighting the alarming potential for exacerbation of water-borne diseases. Floodwaters may inundate, overwhelm or damage water treatment, delivery and waste removal systems, leading to cross-contamination of sewage and drinking water [69]. Over a third of rural Cambodians lack improved water sources, which accounts for over a quarter of the population, meaning that consumption of contaminated water in flood conditions is common [19,69]. Cambodia has the lowest toilet coverage in Southeast Asia, with three quarters of the rural population lacking access to sanitation facilities and most rural villages having no plumbing or electricity [19]. Population displacement and remaining in ‘safe’ areas for more than a few weeks post-flood increases risk of diarrhoeal disease, due to the synergistic effects of malnutrition; overcrowding; increased potential for faecal-oral transmission; and inadequate clean water, sanitation and hygiene [21,68]. Reduced access to sanitation facilities and lack of education about hygiene practices means open defecation is extremely common in Cambodia, utilised by 54% of the population, and 71% of children under five [19]. Changes in water temperature and stagnation have also been implicated in altering diarrhoeal pathogen replication and survival rates [69]. Power shortages related to flooding may disrupt water treatment and supply plants. Emergency delivery of provisions—such as water purification chemicals, soap and water storage containers—is often unable to reach every area of need, for example due to floodwaters blocking roads or damage to civil infrastructure such as bridges [32]. This can also restrict access to health care facilities resulting in treatable conditions such as diarrhoea remaining untreated—this is significant as only 20% of the population have existing access to a health care facility [64].

The recent WHO-Ministry of Health DRIP-SWICCH project (Developing Research and Innovative Policies Specific to the Water-related Impacts of Climate Change on Health), which correlated river height data and rainfall with diarrhoeal disease in Cambodia, demonstrated that unimproved water sources and poor sanitation facilities were the main risk factors for diarrhoeal disease [16]. Similarly, following typhoons, communities relying on uncovered wells and surface water were found to be at heightened risk of diarrhoeal disease, as these sources were most easily contaminated [34,36].

The results of this project suggest that toilet use and piped water may be protective against the effect of flooding on diarrhoeal disease, consistent with findings from the DRIP-SWICCH project [16]. Following the severe 2011 Cambodian floods, children from households with unimproved sanitation facilities and untreated drinking water experienced more diarrhoea than households with treated water and soap [35]. DRIP-SWICCH also provided evidence of a protective effect of education and literacy, particularly for women and girls, against diarrhoeal disease [16], consistent with the 2011 Cambodian floods, whereby diarrhoea incidence was higher in children whose mother lacked an education [35]. Hygiene education is crucial as few households identified water as a potential cause of increased diarrhoea following flooding in Cambodia in 2011 [35].

### 4.3. Limitations

Although there is an abundance of empirical evidence in the international literature of increased incidence of water-borne disease related to extreme weather events such as floods, are results showed more heterogeneous effects of flooding on diarrhoea incidence that expected, with only two provinces demonstrating a statistically significant lagged relationship between flood events and diarrhoeal disease [16,21]. Although a strength of our study was the relatively long time-series analysis with multiple, nation-wide events, significant limitations included the need to define the temporo-spatial parameters of flood events, leading to a potential random measurement error of exposure. Furthermore, less severe cases of diarrhoea would presumably be less likely to be included; we assumed that this bias was consistent across the study period. A related source of bias in our findings lies in the tendency for many Cambodian people to visit private clinics or pharmacies for first-line treatment of many illnesses, including diarrhoea, thus our exclusive use of MoH notification data represents an unknown proportion of the total cases of diarrhoeal disease in the country. Finally, any potential misdiagnoses would potentially affect these study results; again, we assumed that diarrhoeal illness was defined and diagnosed consistently across the study period.

The discrepancy between the literature review and these findings could also be due to inherent inaccuracies of the data and incompleteness of datasets. The true diarrhoea incidence may be under-reported due to financial, technical and/or systemic limitations in the context of an under-resourced, developing country, particularly at times of disaster [25,32,69]. Another possibility is, of course, that such a consistent relationship (*i.e*., between floods and diarrhoeal disease) does not exist in the studied population and the findings are due to chance. It is also possible that the data are accurate and the results are true representations of the relationship between flooding and diarrhoeal disease in these Cambodian provinces, but that other factors mediate the exposure-response relationship, leading to the wide variation in associations between provinces.

Another clear outcome of the literature review conducted is the paucity of country-specific research regarding the impact of extreme weather events on health [25]. Grey literature (such as disaster reports) was predominantly utilised and may be subject to inaccuracies or inconsistencies, highlighting the need for research to elucidate the true incidence of diarrhoeal disease following extreme weather events and the impact of different interventions in Cambodia. Furthermore, the definition of flood, drought and typhoon would have varied in the sources used; this study at least provides an insight into the overall burden of extreme weather events in Cambodia—which was the purpose of this WHO project. 

### 4.4. Future Considerations and Opportunities 

The interplay between flooding and diarrhoeal disease in Cambodia is a very important consideration in the context of the overlapping challenges of health, development and climate change. Increasing frequency and intensity of extreme weather events, projected as a result of climate change in Cambodia, are likely to see an increased risk of diarrhoeal diseases [47,70]. Although uncertainty exists in climate change projections, a 2%–5% increase in the burden of diarrhoeal disease in some low-income countries is predicted to occur by 2020 due to increased heavy precipitation events related to climate change [69,71]. As well as local adaptation, this highlights the urgent need for global reductions in greenhouse gas emissions to mitigate the human toll of climate change. 

This study also highlights the opportunity to combine vulnerability to extreme weather events and climate change; pre-existing health burdens, risk factors and access to health care; and socio-demographic vulnerabilities, to provide an index of climate change and health risk, which currently does not exist. This could help optimize resource allocation according to risk stratification. 

## 5. Conclusions 

Given the inextricable link between human health and the natural environment, human health can be compromised by extreme weather events or the resultant ecological, physical and social disruption. 

Continued global fossil fuel use and the changing climate means that Cambodians are faced with an escalating challenge to overcome the potential health impacts of extreme weather events of unprecedented magnitude or duration. Chief among these will be water-borne or water-related diseases, such as diarrhoeal illness—one of the preeminent health burdens in development countries. It is hoped that the findings of this research will help to guide public health and community interventions to protect the health of Cambodian communities from these detrimental health impacts. 

Further research into the health impacts of extreme weather events, vulnerabilities and early warning and response tools is urgently required. Tailoring adaptation policies, resources and interventions to the local drivers of vulnerability is vital to strengthen adaptive capacity and resilience of the Cambodian population.
